# A PSO-based energy-efficient data collection optimization algorithm for UAV mission planning

**DOI:** 10.1371/journal.pone.0297066

**Published:** 2024-01-19

**Authors:** Lianhai Lin, Zhigang Wang, Liqin Tian, Junyi Wu, Wenxing Wu

**Affiliations:** 1 School of Computer Science, Qinghai Normal University, Xining, Qinghai, China; 2 School of Computer Science, North China Institute of Science and Technology, Langfang, Hebei, China; TU Wien: Technische Universitat Wien, AUSTRIA

## Abstract

With the development of the Internet of Things (IoT), the use of UAV-based data collection systems has become a very popular research topic. This paper focuses on the energy consumption problem of this system. Genetic algorithms and swarm algorithms are effective approaches for solving this problem. However, optimizing UAV energy consumption remains a challenging task due to the inherent characteristics of these algorithms, which make it difficult to achieve the optimum solution. In this paper, a novel particle swarm optimization (PSO) algorithm called Double Self-Limiting PSO (DSLPSO) is proposed to minimize the energy consumption of the unmanned aerial vehicle (UAV). DSLPSO refers to the operational principle of PSO and incorporates two new mechanisms. The first mechanism is to restrict the particle movement, improving the local search capability of the algorithm. The second mechanism dynamically adjusts the search range, which improves the algorithm’s global search capability. DSLPSO employs a variable population strategy that treats the entire population as a single mission plan for the UAV and dynamically adjusts the number of stopping points. In addition, the proposed algorithm was also simulated using public and random datasets. The effectiveness of the proposed DSLPSO and the two new mechanisms has been verified through experiments. The DSLPSO algorithm can effectively improve the lifetime of the UAV, and the two newly proposed mechanisms have potential for optimization work.

## 1 Introduction

With the rapid development of the Internet of Things (IoT), more and more devices are connected to the Internet [1]. Among them, UAVs, as a link in the IoTs, have a broad application prospect [2–4]. With the arrival of the Industry 4.0 era, sensing technology and UAV driving technology have been developed rapidly, which makes UAVs more widely used in agriculture, forestry, and other fields [5–7].

However, an important question is faced after the large-scale deployment of IoT devices: how to efficiently collect the data generated by these devices [8]? Since many IoT devices are distributed in long-distance or inaccessible areas and have limited energy supply, traditional data collection methods can no longer meet the demand. Therefore, we need to develop novel data collection methods to solve this challenging task [9, 10].

Some novel data collection schemes have already emerged. For example, the use of mobile base stations or satellite networks for data collection, the use of technologies such as low-power Bluetooth to achieve miniaturized transmission in a localized range, and the improvement of the accuracy and stability of UAV flights by carrying artificial intelligence algorithms and autonomous navigation systems [11, 12]. These programs provide new solution directions for solving data collection problems.

In recent years, the use of UAVs to accomplish data collection tasks has become a popular topic. First, due to UAV mobility and flexibility, UAVs can move freely in a variety of environments and can quickly reach the target location for data collection. Second, in terms of establishing a line-of-sight link with the target device, UAVs ensure a stable communication connection for efficient data transmission. In addition, UAVs can provide emergency services for IoT devices during temporary or unexpected events, such as emergency rescue and monitoring [13].

In sensor networks, energy consumption is one of the most important factors affecting the lifetime of the device [14]. Many IoT devices have limited energy and are rarely recharged. Therefore, reducing energy consumption is crucial to extending the device’s lifetime.

Among these studies mentioned above, this paper, on the other hand, focuses on how to improve mission planning during UAV data collection and use optimization algorithms to arrange the stopping points of the UAVs to reduce the total energy consumption of the UAVs as well as the IoT devices.

Based on the application background and with reference to the PSO optimization algorithm, this paper proposes an improved swarm intelligence algorithm. The algorithm is able to achieve a better balance between global and local search. Since we believe that its individual motion process has a certain degree of similarity with the particle swarm, we refer to it as an improved PSO algorithm, and the contributions of this paper are as follows:

1. Noting that the current problem is a position-finding problem, the PSO is used as the basis for the optimization, and the PSO’s movement method is applied to the optimization algorithm (the background of the PSO originates from the observation of bird foraging behaviors, which is in line with the characteristics of the current problem in terms of the position-finding).
2. Since the number of UAV stopping points is not fixed, a variable population strategy is designed for the PSO optimization algorithm.
3. We notice that the local search capability of the simple PSO is inadequate, from therefore we propose a Self-Limiting Radius (SLR) mechanism in this paper to compensate for the lack of local search capability of the algorithm.
4. In addition, the global search capability of PSO is also inadequate compared to other current algorithms, so we propose a Multiple Simulated Annealing (MSA) strategy to enhance the global search capability. And we conduct experiments to test the performance of MSA.
5. The DSLPSO algorithm is finally realized, which achieves a better-balanced effect in global and local search.

**Fig 1:**
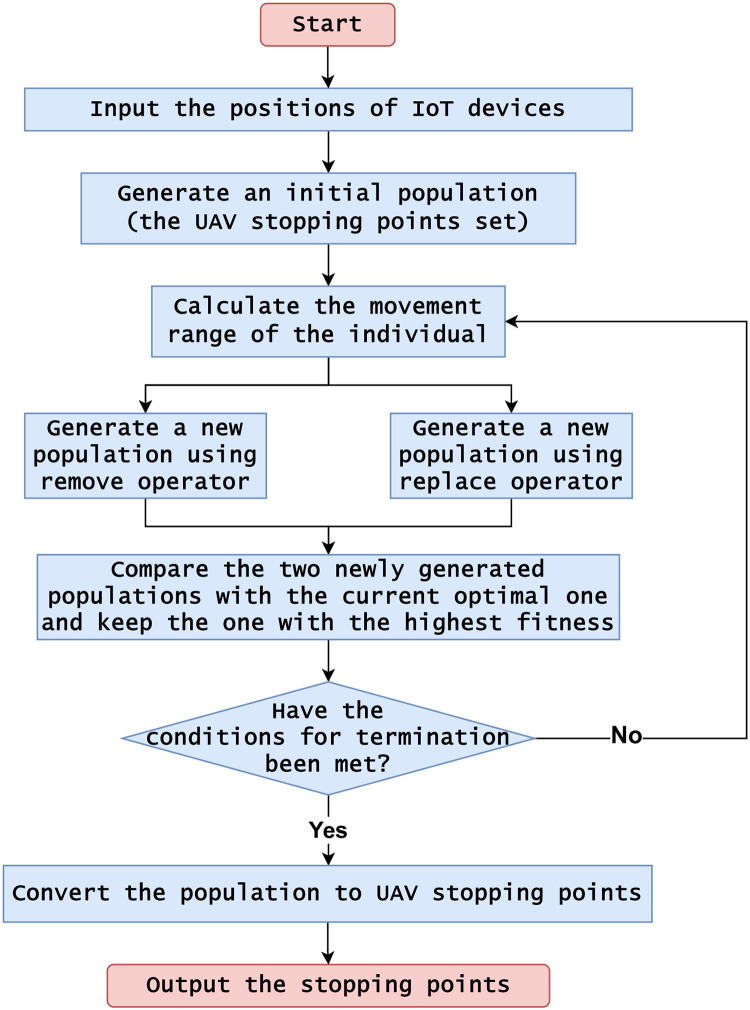
The workflow diagram of DSLPSO.

The workflow diagram of DSLPSO is shown in Fig 1.

The rest of the paper is organized as follows. Section 2 describes progress on current research issues over the past two years. Section 3 is about materials and methods. Section 3.1 describes the energy consumption formulation of the UAV-based IoT data collection system. Section 3.2 describes the framework and implementation of DSLPSO. Section 4 gives experimental results and discussion. Section 5 concludes with final remarks and further work.

## 2 Related studies

Despite the great advantages of UAVs in assisting data collection for the IoT network, how to improve their efficiency remains a key issue to be addressed. In recent years, scholars have turned their attention to UAV deployment optimization and flight trajectory planning [15]. Recently, reference [16] gave a more comprehensive review of UAV-based sensing networks.

Reference [17] introduces the DEVIPS (differential evolutionary algorithm for variable population sizes) algorithm. DEVIPS optimizes the deployment of UAVs by considering the number and location of UAV stopping points in an IoT data collection system. The method demonstrates the potential of evolutionary algorithms to solve variable-length optimization problems, and the paper defines a standard UAV-based data collection system, so it has been studied by many scholars.

In the last two years many scholars joined the research in this direction, reference [18] proposed a new population-based optimization algorithm BSADP(backtracking search algorithm with dynamic population). This algorithm solves the energy consumption problem of the UAV-based IoT data collection system by determining the optimal number and location of UAVs’ stopping points. The BSADP provides a simple framework combining an improved backtracking search algorithm and a population based on the inverse learning adjustment mechanism. Reference [19] proposed an enhanced energy-efficient data collection optimization algorithm for UAV clusters in IoT. The algorithm focuses on reducing the total energy consumption while optimizing the number and location of UAVs. By considering the relationship between energy harvesting and energy consumption, the data collection efficiency is significantly improved. Reference [20] proposed a UAV-based IoT data collection mechanism for low-latency data delivery in sparse deployment scenarios. The mechanism aims to overcome the data transmission bottleneck from the edge region of the ground sensor network to the base station. By utilizing a cooperative relay system, the approach improves the age of information performance in UAV-based IoT data collection.

In addition, reference [21] proposed a memetic algorithm based on isomorphic transcoding space to optimize the deployment of UAVs, especially to solve the problem of UAV distribution in energy-efficient artificial intelligence of things data collection. Reference [22] addresses the use of height information of UAVs for multi-source localization in the efficiency problem, a sound source localization model compatible with PSO is proposed. Reference [23] considers UAV-enabled wireless powered communication networks scenarios where UAVs need to cover ground-based wireless devices, a non-dominated sequential genetic algorithm with improved K-means initialization and variable dimensionality mechanism is proposed to solve the power and trajectory optimization problem for UAVs. Reference [24] proposed an adaptive trajectory optimization algorithm to minimize the energy consumed by mobile edge computing and minimize the process urgency indicator. Reference [25] employs a variable length trajectory planning algorithm, which includes a genetic algorithm to update the stopping point deployment and deal with the problem of associating UAVs with stopping points and user devices. Reference [26] proposed a joint deployment and trajectory optimization framework for UAV applications in IoT systems. UAV deployment optimization is performed by introducing an adaptive whale optimization algorithm and UAV flight trajectory optimization by introducing elastic ring self-organizing mapping. Reference [27] proposed a multi-objective trajectory optimization algorithm based on cutting and padding coding strategy to minimize the energy consumption and task urgency of a single UAV-based mobile edge computing system, which provides computing services for ground-based IoT devices using UAVs, and the algorithm performs well in the validation experiments. Advanced metering infrastructure for smart meter data collection via UAVs is investigated in reference [28], the total cost of electricity is minimized by jointly optimizing the number of UAVs, power supply size, charging location, and data collection trajectory planning. Reference [29] proposed a method to jointly optimize UAV flight trajectories and passive phase shifts of intelligent reflecting surfaces to save energy consumption and task completion time for multiple UAVs.

The above mainly summarizes the research in the direction of UAV-based data collection in the past two years, many of which refer to the theoretical research in the reference [17], and methods such as swarm intelligence and neural networks are current research hotspots in this direction. In addition to planning the trajectory of the UAV to reduce energy consumption, there are also studies on the latency of the sensing network [30], sensor lifetime [31], and sensing protocols [32] to ensure secure data transmission.

In the current research on UAV-based data collection systems, genetic algorithms or swarm intelligence algorithms are used, but we found that genetic algorithms (e.g., genetic algorithm, differential evaluation algorithm) do not take into account that the problem is a position-finding problem, so these algorithms are ineffective in terms of local optimization, and algorithms based on swarm intelligence (e.g., dandelion algorithm, PSO) do not have a strong global search capability for the current problem, and to solve the problem we have proposed the DSLPSO algorithm, which strengthens the algorithm’s ability of local searching.

## 3 Materials and methods

### 3.1 Problem formulation

**Fig 2:**
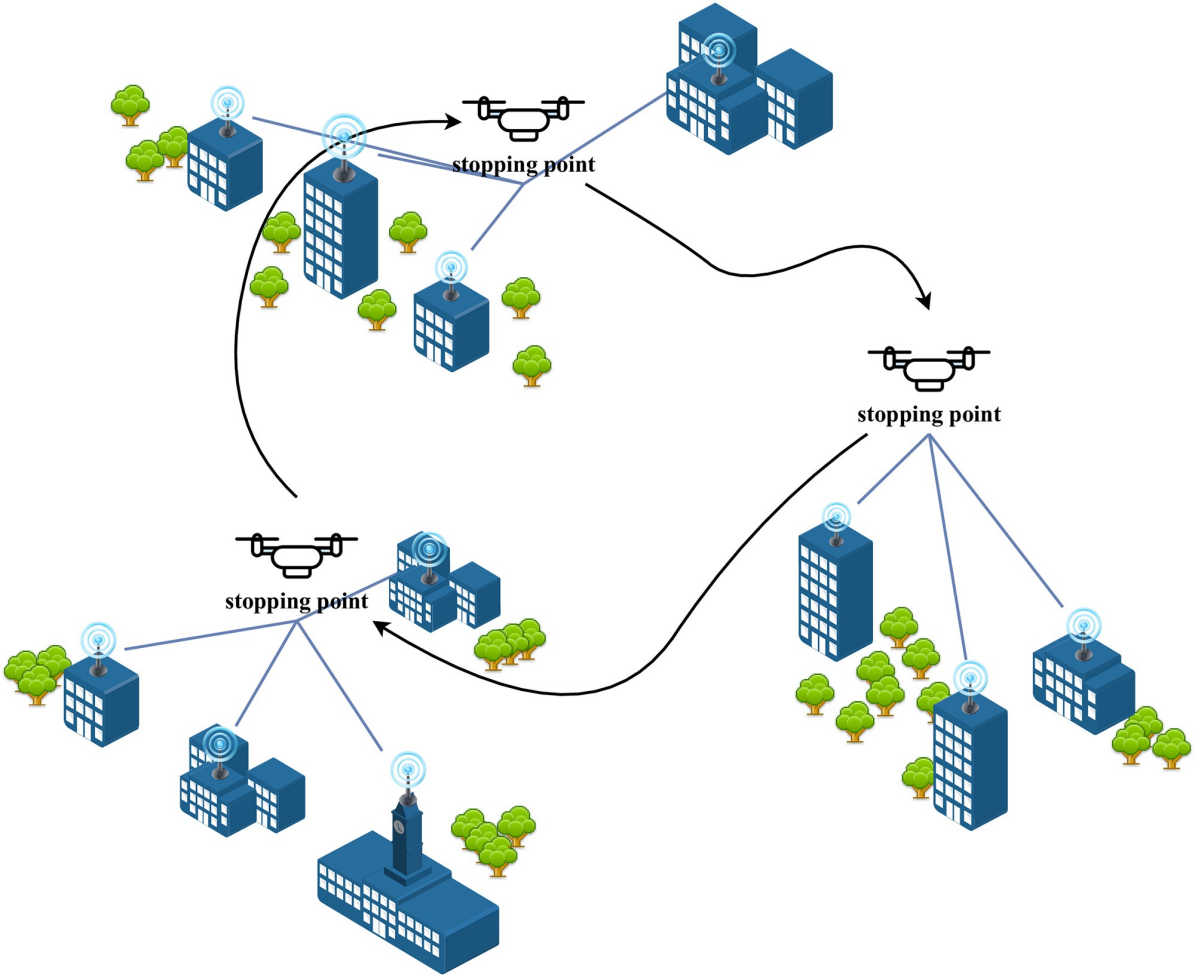
Data collection system with a single UAV.

As shown in Fig 2, a UAV-based IoT data collection system. In this system, the UAV flies at a fixed altitude in order to collect data from the area at each stopping point. By flying between each stopping point, data collection for the whole area is eventually realized. In our study, we will focus on the energy consumption of data transmission and UAV hovering in the system (after the stopping points are found, the problem will be transformed into a typical traveling salesman problem for a single UAV data collection system, and thus the flight energy consumption can be further solved using the traveling salesman problem solution method, as in the reference [15]).

The design and optimization of UAV-based data collection systems are of great significance in many fields such as environmental monitoring and agricultural observation. Energy consumption can be reduced and the efficiency of data collection can be improved through rational planning of UAV routes and flight strategies.

In this study, we will evaluate the energy consumption of the system by comprehensively considering the UAV’s hovering energy consumption and transmission energy consumption during data collection.

Since the UAV is free in 3D space and are not affected by ground structures, it is free to choose stopping points within the mission area of data collection. Considering the mission area as a 3D space and assuming that there are *m* IoT devices in the space. The location of the *i*th(*i* ∈ [1, *m*]) IoT device is (*x*_*i*_, *y*_*i*_, 0). Assume that the UAV flies at a constant altitude of *H* and the number of stopping points of the UAV is *n*, then the location of the *j*th(*j* ∈ [1, *n*]) UAV stopping point is (*X*_*j*_, *Y*_*j*_, *H*). The distance between the stopping point and the IoT device can be expressed as [24]:

$$\begin{array}{c} d_{ij}=\sqrt{{\left(X_{j}-x_{i}\right)}^{2}+{\left(Y_{j}-y_{i}\right)}^{2}+{\left(H-0\right)}^{2}} \end{array}$$ (1)

A binary variable *C*_*ij*_ is used to indicate the correspondence between a UAV and an IoT device, where *C*_*ij*_ = 1 indicates that a data connection is established between the *i*th IoT device and the *j*th UAV stopping point, and *C*_*ij*_ = 0 indicates that no data connection is established. A UAV can only support data transmission of *M* IoT devices at the same time.

Then *C*_*ij*_ needs to satisfy the following constraints [24]:

$$\begin{array}{cc} & C1:C_{ij}\in \left\{0,1\right\} \end{array}$$ (2)

$$\begin{array}{cc} & C2:\sum_{j=1}^{n}C_{ij}=1 \end{array}$$ (3)

$$\begin{array}{cc} & C3:\sum C_{ij}=m \end{array}$$ (4)

$$\begin{array}{cc} & C4:\sum_{i=1}^{m}C_{ij}\leq M \end{array}$$ (5)

The energy consumption between the *i*th IoT device and the *j*th UAV stopping point during data transmission is [17]:

$$\begin{array}{c} G_{ij}=G_{0}d_{ij}^{-2} \end{array}$$ (6)

where *G*_0_ denotes the channel gain at a distance of 1 meter. Thus the data rate can be expressed as [17]:

$$\begin{array}{c} R_{ij}=B\;{\text{log}}_{2}\;\left(1+\frac{P_{i}G_{ij}}{\delta^{2}}\right) \end{array}$$ (7)

where *P*_*i*_ is the transmit power between the *i*th IoT device and the *j*th UAV stopping point, *B* is the system bandwidth, and *δ*^2^ is the white Gaussian noise power.

Let *E*_*ij*_ denote the energy consumed to send the data volume *D*_*i*_ between the *i*th IoT device and the *j*th UAV stopping point. Then [18]:

$$\begin{array}{c} E_{ij}=\frac{P_{i}D_{i}}{R_{ij}} \end{array}$$ (8)

Then all the energy consumption *E*_*IoT*_ generated by data transmission can be expressed as [18]:

$$\begin{array}{c} E_{IoT}=\sum_{i=1}^{m}\sum_{j=1}^{n}C_{ij}E_{ij},i\in \left[1,m\right],j\in \left[i,n\right] \end{array}$$ (9)

Since we assume that the UAV hovers over the stopping point until it completes the data transmission task at that point, the hovering time *T*_*j*_ of the UAV at the *j*th stopping point is [15]:

$$\begin{array}{c} T_{j}=\text{max}\;\left(C_{ij}\frac{D_{i}}{R_{ij}}\right) \end{array}$$ (10)

The total energy consumed by the UAV hovering is [15]:

$$\begin{array}{c} E_{UAV}=\sum_{j=1}^{n}P_{h}T_{j} \end{array}$$ (11)

where *P*_*h*_ is the hovering power of the UAV.

Based on the above description, the energy consumption of the whole UAV data collection process can be defined as [15, 17, 18]:

$$\begin{array}{c} \underset{\{X_{j},Y_{j}\},n}{\text{min}}E_{UAV}+\epsilon E_{IoT},j\in \left[1,n\right] \end{array}$$ (12)

where *ϵ* is the weight between the energy consumption of the UAV and the energy consumption of all IoT devices.

And this optimization needs to satisfy the constraints C1 to C4 as well as C5 to C8 constraints [18]:

$$\begin{array}{cc} & C5:X_{min}\leq X_{j}\leq X_{max} \end{array}$$ (13)

$$\begin{array}{cc} & C6:Y_{min}\leq Y_{j}\leq Y_{max} \end{array}$$ (14)

$$\begin{array}{cc} & C7:H_{min}\leq H\leq H_{max} \end{array}$$ (15)

$$\begin{array}{cc} & C8:n_{min}\leq n\leq n_{max} \end{array}$$ (16)

where *X*_*min*_ and *X*_*max*_ denote the upper and lower bounds of *X*_*j*_, respectively. *Y*_*min*_ and *Y*_*max*_ denote the upper and lower bounds of *Y*_*j*_, respectively. *H*_*min*_ and *H*_*max*_ denote the upper and lower bounds of *H*, respectively. *n*_*min*_ and *n*_*max*_ denote the upper and lower bounds of *n*, respectively.

### 3.2 Proposed algorithm

We note that many scholars currently use evolutionary algorithms such as genetic algorithms and differential evolution algorithms to solve the data collection problem of UAVs, and the current problem is a problem of position-finding, evolutionary algorithms due to the mechanism of the later fine-tuning of the stopping point is relatively inadequate, such as the algorithm of [17] is very good at searching globally, but the search ability is not strong in the local range, the algorithm’s solution may not be a locally optimal solution due to the lack of a process to fine-tune the optimization result. (We will show this in Section 4.4)

Although swarm intelligence algorithms such as [18] take into account the characteristics of the problem and can better balance the local convergence of the algorithm at the later stage, in practice, we find that the sowing radius mechanism of the algorithm in [18] changes too fast, the global search ability cannot meet the requirements of the problem, and it is easy to fall into the local optimum, and it is difficult to jump out of such a dilemma, so we hope to propose a new mechanism, SLR, to replace the sowing radius.

PSO algorithm originates from the observation of bird foraging behavior, which is essentially a position-finding method, and its application background is similar to the UAV stopping point position-finding, meanwhile, the PSO has fewer parameters and is simple to implement, so it is easy to be extended and modified, so we consider the PSO as the basic algorithm, and the SLR to improve the algorithm’s local search capability, and after several experiments, we determined that the current problem has high requirements for the global search ability of the optimization algorithm, and based on several experiments, we proposed the MSA to improve the global search ability of the algorithm, and in the end, we proposed a new variable population mechanism to meet the solution conditions of UAV data collection problem.

#### 3.2.1 Variable population strategy

Since the DEVIPS study used the variable population strategy, the variable population approach has received some attention in the UAV data collection problem, and the key idea is to turn “each individual corresponds to a solution of the problem” into “the whole population corresponds to a solution of the problem.

In the current research problem, since the number of stopping points of UAVs is uncertain, assuming that in the current UAV mission planning problem, the data collection range is 1000 × 1000 (*X*_*max*_ = *Y*_*max*_ = 1000), the number of IoT devices is 100, and the number of simultaneous communication supported by UAVs is 5, the number of stopping points usually fluctuates between 20 and 30 after solving the problem with the optimization algorithm, so it is difficult to determine the coding method of the individuals.

**Fig 3:**
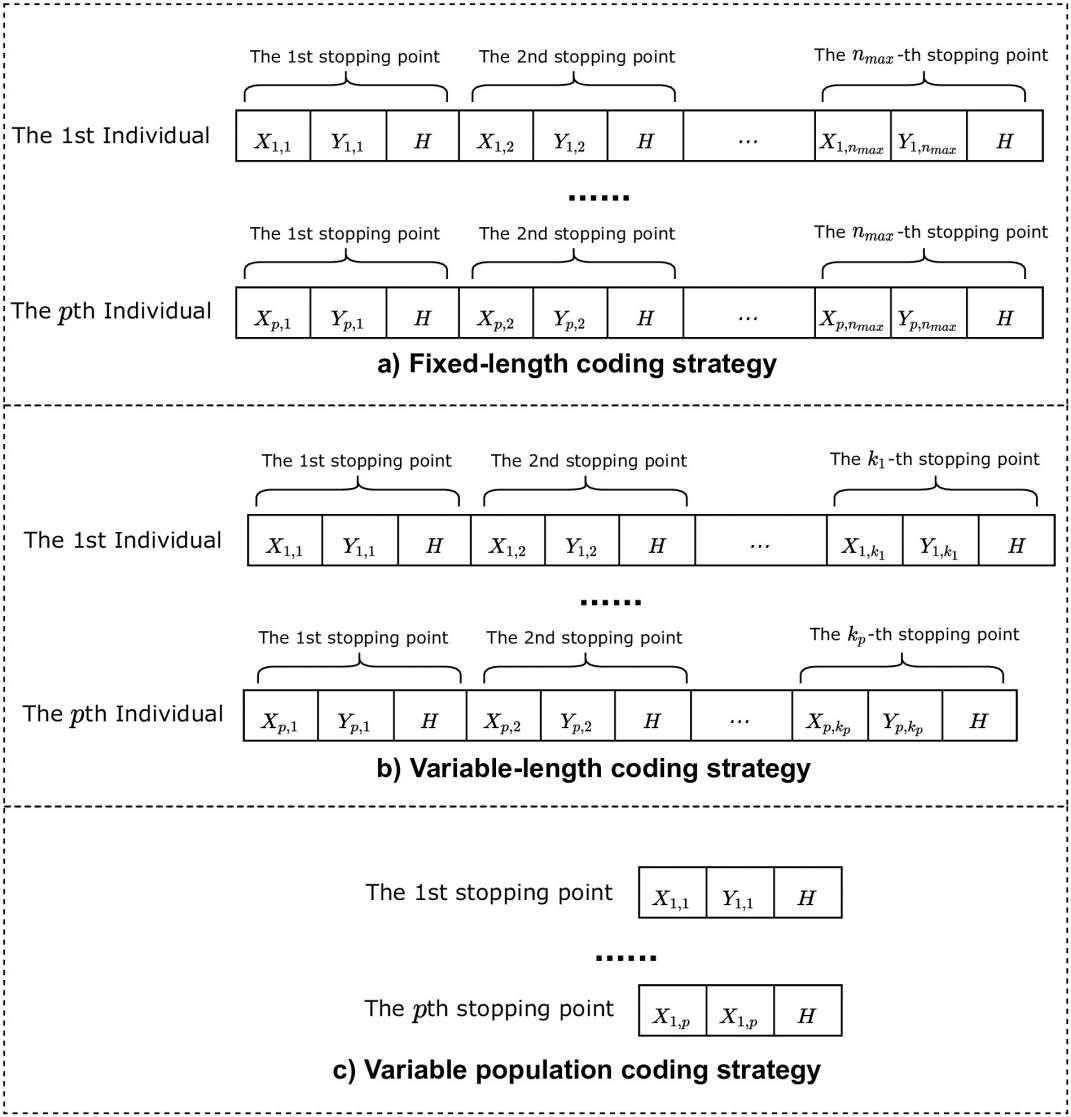
Three coding strategies commonly used in UAV data collection mission planning.

As shown in Fig 3, there are three common coding strategies.

The first one is the fixed-length coding method, under which an individual represents a solution to a problem, but the fixed-length coding method faces the following problems: The coding length of an individual will be taken as “the dimension of the problem × the maximum number of stopping points”, i.e. 3 × 100 for the standard problem mentioned above, which makes the coding of an individual particularly long and increases the computational complexity. Furthermore, the probability of generating better subindividuals when using the population updating algorithm will be reduced due to the long coding length.

The second is the variable-length coding strategy, in which the coding length is not fixed, and each individual may have a different coding length, which ensures that the algorithm can search for a different number of stopping points, but at the same time, due to the different coding lengths of each individual, it is difficult to achieve evolutionary iteration between individuals.

The third is the coding strategy of variable population, that is, one of the current hot research algorithms, references [15, 17, 18], and many other papers have adopted this method. In this coding strategy, each individual represents the coordinates of a stopping point, while the whole population represents one deployment.

In this paper, the third coding strategy is adopted because it does not require the design of special crossover and mutation operators and does not cause the search chaos problem, and each individual contains only three dimensions, so the evolutionary iteration between individuals can be simply realized.

#### 3.2.2 Basic framework of DSLPSO

Algorithm 1 demonstrates the basic flow of DSLPSO, as can be seen from that, the DSLPSO algorithm can be divided into two phases, the initialize phase and the iterative phase: the first phase is to initialize the population using the random initialization method (When using the random initialization method to generate the initial population, if the generated population does not satisfy the constraints, it is randomized again until the constraints are satisfied), and then enter into an iterative phase, in which the SLR is updated according to the current number of iterations, and then computed using two operators, remove and replace.

**Algorithm 1** The proposed DSLPSO

**Input:** The positions of IoT devices

**Output:** The positions of UAV stopping points

1: Generate an initial population using random initialization methods

2: **for** *fe* < *feMax* **do**

3:  Calculate the SLR.

4:  Calculate the fitness of the population, updating populations using the remove operator

5:  7Calculate the fitness of the population, updating populations using the replace operator

6: **end for**

7: Convert the population to UAV stopping point positions

Where *fe* denotes the number of runs of the fitness evaluation function and *feMax* is the maximum number of runs of the fitness evaluation function, and the fitness of the population is the total energy consumption of the UAV data collection process. In the framework of this algorithm, we refer to DEVIPS using the *fe* as the termination flag of the algorithm, to verify whether this metric can reflect the actual computational complexity (see Section 4.2 Table 4) of the algorithm, we tested the algorithm’s time consumption in the various processes in the experiment section, and found that the algorithm spends about 90% of the time in the population fitness evaluation, so it is reasonable to adopt *fe* as the variable for the termination condition.

In the fitness evaluation function, the computational effort is mainly in calculating the distance between the stopping point and the IoT devices, and since the number of stopping points is positively correlated with the number of devices, the complexity of calculating the distance is *O*(*m*^2^), and the time complexity of the algorithms proposed in this paper is in line with similar algorithms based on the use of *fe* as the variable for the termination condition, which is *O*(*m*^2^ × *feMax*).

### 3.3 SLR mechanism and MSA strategy

Since the simple motion of the standard PSO, the local optimization effect is inadequate, in order to solve this problem, we propose the mechanism of SLR. The basic principle of SLR is: when generating sub-populations in each iteration, the subindividuals are always generated in a circle with a certain radius, the self-limiting radius, and centered on the previous generation. In this paper, we use *vslr* to denote the value of the self-limiting radius and SLR to denote the self-limiting mechanism.

We believe that the use of SLR can increase the motion effectiveness of PSO, and can effectively limit the problem of over-speed of the individuals of PSO when they move. Meanwhile, in order to ensure that the motion nature of PSO is not affected, some modifications are also made to the definition of particle velocity. In DSLPSO, the particle’s velocity is defined as follows: the initial velocity of the individual is set to 0, if the next generation of the individual’s fitness is better than the current individual, then the velocity of the next generation is set to the distance between the position of the next generation and the current individual’s position, and if the next generation of the individual’s fitness is not as good as the current individual, then the velocity of the next generation individual is set to 0.

On this basis, we define the movement mode of the particle: if the velocity of the current individual is not 0, it moves *vslr* meters in the direction of the velocity, and if the velocity of the current individual is 0, it moves randomly in a circle of radius *vslr* centered on the current individual’s position. In addition, the updating of the *vslr* is also an important issue, and to solve this problem, we refer to the simulated annealing method and improve it by proposing the MSA, MSA uses a multiple simulated annealing algorithm for *vslr* updating. In the proposed MSA mechanism, *vslr* is updated as follows:

Let the number of simulated annealing be *qtime* and the total number of iterations be *feMax*, then an annealing has a total of *cUnit* = *feMax*/*qtime* generations, let the maximum *vslr* be *R*_*max*_ (this value is usually equal to *X*_*max*_), and the current generation is *fe*, then the *vslr* can be calculated by the following equation:

$$\begin{array}{c} vslr=\frac{R_{max}}{2^{\left\lfloor \frac{fe}{cUnit}\right\rfloor }}\times \frac{(feMax-fe)\%cUnit}{cUnit} \end{array}$$ (17)

where ⌊*A*⌋ denotes rounding down to *A* and % denotes the remainder operation. The equation is divided into two parts by the multiplication sign, the first half is used to compute the maximum *vslr* of the current simulated annealing process, and the second half is used to compute the progress of the current iteration in the current simulated annealing process.

**Fig 4:**
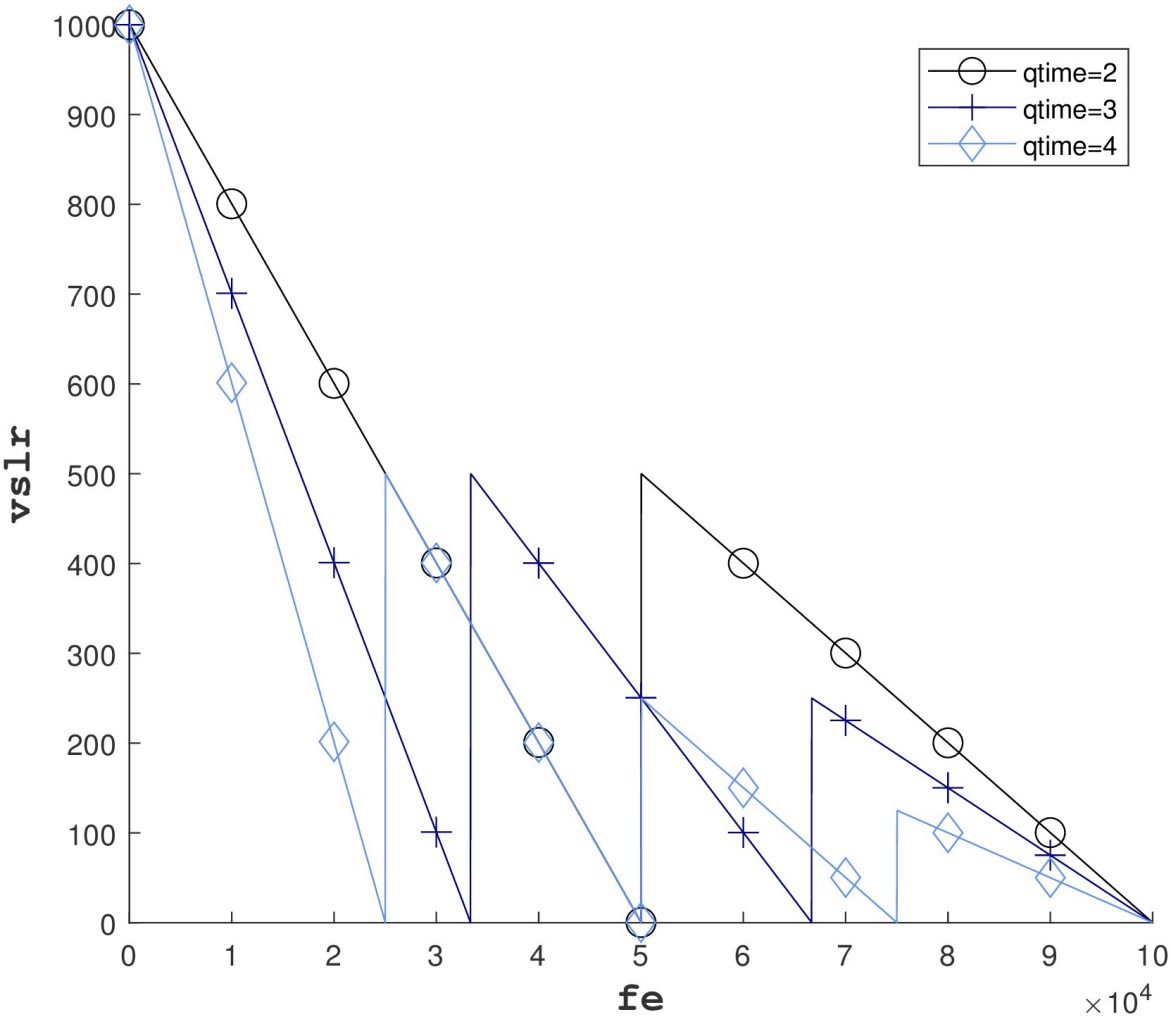
*vslr* during iteration.

The algorithm operates in practice with the variation of *vslr* versus *qtime* as shown in Fig 4.

Under the control of SLR mechanism and MSA strategy, the range of the individual’s movement is limited by *vslr* and at the same time, *vslr* is limited by MSA, i.e., there are two mechanisms for limiting the movement of an individual, so we call this PSO algorithm the double self-limiting PSO algorithm.

#### 3.3.1 The replace and remove operators

Inspired by [17], we propose a novel variable population strategy.

Usually, the operator of the variable population contains three operators, the insert operator, the remove operator, and the replace operator, but we consider the characteristics of PSO and use two operators, the remove operator and the replace operator, so that the operators are more in line with the form of PSO.

The remove operator is used to reduce the number of UAV stopping points to an optimal number, we found during our experiments that the remove operator usually only plays a role in the early stage, and does not participate in the optimization in the later stage, for this reason we designed a self-adaptive remove operator, see Algorithm 2 for the algorithm description.

**Algorithm 2** Remove operator

**Input:** The population of DSLPSO (representing all UAV stopping points)

**Output:** Population of DSLPSO after deletion of some individuals

1: *rr* = 0;

2: *rc* = 1;

3: **if** *rc* > 0 **then**

4:  *rf* = 0;

5:  **for** *i*= 1 to N **do**

6:   Remove the *i*th individual from the population, noting that the current population is *X*_*r*_

7:   Compute the fitness of the *X*_*r*_ population, denoted as *fval*

8:   **if** *fval* < *Fbest* **then**

9:    *rf* = 1;

10:    *rr* = 0;

11:    Replace the current population with *X*_*r*_

12:   **end if**

13:  **end for**

14:  **if** *rf* == 0 **then**

15:   *rr* = *rr* + 1;

16:   *rc* = *rc* − *rr*;

17:  **else**

18:   *rc* = *rc* + 1;

19:  **end if**

20: **else**

21:  *rc* = *rc* + 1;

22: **end if**

In the remove operator, the population size is assumed to be *N*, we control the timing of the remove operator using three variables, *rr* (to record the number of consecutive failures of the remove operator), *rc* (the number of iterations until the next remove operation), and *rf* (whether or not this iteration produces an optimization), in this way, in the early stages of the algorithm, the remove operator will normally reduce the number of stopping points, while in the later stages, the remove operator will significantly reduce the number of runs and the amount of computation.

The replace operator is the core of the DSLPSO, In this section, we will implement the way the particles move under the SLR mechanism, whose pseudo-code descriptions are given in Algorithm 3.

**Algorithm 3** Replace operator

**Input:** The population of DSLPSO (representing all UAV stopping points)

**Output:** Population of DSLPSO after individual movement

1: **for** *i* = 1 to N **do**

2:  ind = P{*i*}

3:  **if** ind.*v* ≠ 0 **then**

4:   indn = ind + rand(1, 3) ×*SLR*;

5:  **else**

6:   indn = ind + ind.*v*;

7:  **end if**

8:  *fval* = Fitness(P)

9:  **if** *fval* < *Fbest* **then**

10:   *v*_1_ = indn—ind;

11:   *v*_2_ = sqrt(sum(*v*_1_ × *v*_1_) / sqrt(sum(*SLR* × *SLR*)));

12:   indn.*v* = *v*_2_;

13:  **else**

14:   indn.*v* = 0;

15:  **end if**

16:  P{i} = indn;

17: **end for**

Where the population size is assumed to be *N*, rand(1,3) denotes three random numbers from 0 to 1, forming a vector, Fitness is the fitness evaluation function, “×” means that the elements of the corresponding dimension are multiplied to form a new vector.

In order to facilitate the understanding, this paper uses ind.*v* to denote the current velocity of the ind particle (For the actual implementation, we put the velocity after the individual coding, i.e., the actual coding of each individual has five dimensions).

The replace operator contains two parts: individual iteration and velocity update. In the individual iteration part, each individual moves according to the current velocity: if the velocity is not 0, it moves according to the velocity, and if no optimization has been generated in the previous generation and the velocity is 0, the individual moves randomly within the *vslr*.

In the velocity update part, the population fitness after the iteration change is first calculated. If no optimization is produced, the velocity of the current individual is set to 0, and this individual is allowed to move randomly in the next iteration. If an optimization is produced, the direction vector of the velocity is calculated, so that the modulus of this vector is equal to *vslr* and set to the velocity of the individual.

## 4 Results and discussion

In this section, we design four experiments to answer the following four questions:

1. Does SLR and MSA proposed in this paper improve the optimization of the algorithm?
2. Does DSLPSO have an advantage over other algorithms in the current UAV mission planning problem?
3. Does DSLPSO still work well for the current mission planning problem with different data collection ranges and number of IoT devices, and is the DSLPSO algorithm generalizable enough?
4. In the first three experiments, we set *qtime* (the number of annealing) of the DSLPSO to 2 by default, the SLR is affected by the change of *qtime* and the number of iterations, so how should we choose *qtime*? How does the MSA play a role in optimization?

### 4.1 Experimental environment

The parameters of the experiment are shown in Table 1, which are basically the same as [17, 18].

**Table 1:** Experimental environment.

Parameter	Symbolic	Value
Number of IoT	*m*	[100, 700]
Maximum value of X-axis	*X*_*max*_	[1000, 3000]m
Maximum value of Y-axis	*Y*_*max*_	[1000, 3000]m
Flight altitude of UAVs	*H*	200m
Height of IoT devices	-	0
Amount of data	*D*_*i*_	[1, 1000]MB
Number of simultaneous transmissions supported by UAV	*M*	5
Number of evaluation function calls	*feMax*	[1, 6] × 10^5^
Transmission power	*P*_*i*_	0.1W
Channel gain at 1m	*G*_0_	-30dB
White Gaussian noise power	*δ* ^2^	-250dBm
Bandwidths	*B*	1MHz
Number of annealing times	*qtime*	[2, 6]
Hover power of UAV	*P*_*h*_	1000W

All experiments were run on MATLAB (2021b), Windows 10 operating system (64-bit), 16G of operating memory.

### 4.2 Impact of MSA and SLR

In order to verify whether our proposed SLR and MSA are effective, in this experiment, we used three different PSO-based algorithms, see Table 2, which are all PSOs that we have improved according to the variable population strategy, among which SAPSO (self-adaptive PSO) can be regarded as the one-time simulated annealing DSLPSO, the VPPSO is a PSO algorithm with a variable population strategy. In brief, SAPSO uses SLR mechanism on the basis of VPPSO, and DSLPSO uses MSA strategy on the basis of SAPSO.

**Table 2:** Three PSO algorithm proposed.

Algorithm	Full Name	Comment
DSLPSO	Double Self-Limiting PSO	The main algorithm studied in this paper.
SAPSO	Self-Adaptive PSO	The difference from DSLPSO is that the SLR uses a traditional adaptive strategy (i.e., linearly decreasing with the number of iterations).
VPPSO	Variable Population PSO	A PSO Algorithm with variable population.

With this experiment, we hope to answer the following two questions:

1. Has the introduction of the SLR mechanism in optimization improved the effectiveness of the algorithm? (By comparing the performance of SAPSO and VPPSO)
2. How does the MSA strategy affect the optimization process? (By comparing the performance of DSLPSO and SAPSO)

**Fig 5:**
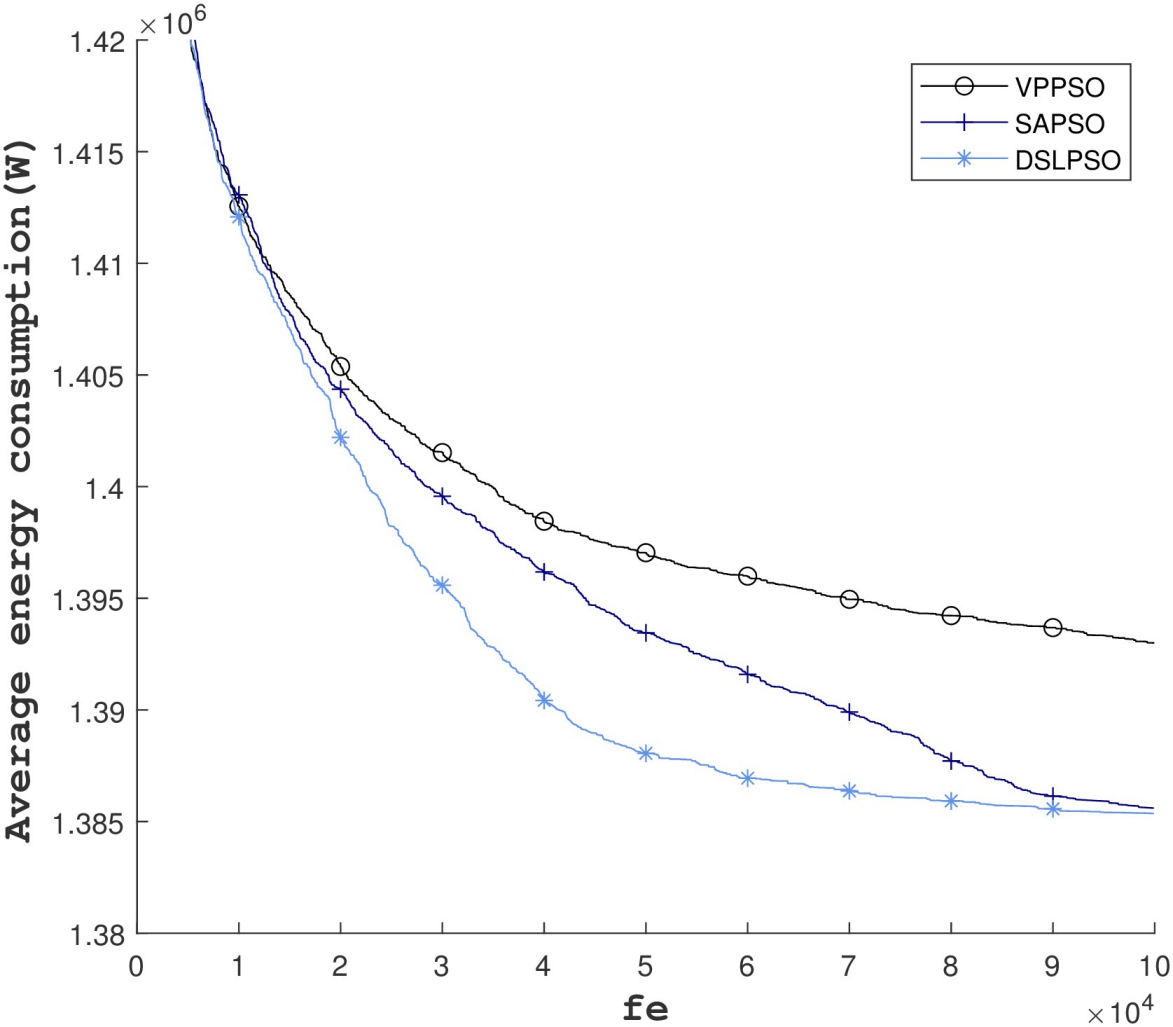
Results of 100 runs of the three PSO-based algorithms.

We let each algorithm be executed repeatedly for 100 times, and then the average of each generation is counted to plot the figure. The results of the three algorithms are shown in Fig 5. It can be seen that the DSLPSO algorithm performs the best, followed by SAPSO, and in the final result, DSLPSO wins by a narrow margin, and the VPPSO algorithm performs relatively general, but if we look at the overall energy consumption, the three algorithms end up with results differing by around 1%, and we believe that all three algorithms are efficient algorithms.

A detailed numerical comparison of the three algorithms is shown in Table 3:

**Table 3:** Energy consumption comparison of the three proposed PSO algorithms.

	VPPSO	SAPSO	DSLPSO
mean	1.3930E+6	1.3856E+6	1.3854E+6
std	6.0972E+3	6.5423E+3	6.4521E+3

Through the iteration figure and statistical results, we believe that: the VPPSO can converge faster when the number of iterations is small, but it can not converge further in the later stage because there is no restriction and the particles wander randomly. The SAPSO algorithm, which is a one-weighted self-limiting algorithm, has a smoother convergence process and the overall iteration is better than VPPSO. The DSLPSO algorithm the algorithm has a good convergence effect in the early stage. It converges further in the later stage, which guarantees the overall convergence effect and is the best-performing one.

Finally, to verify the reasonableness of using the number of evaluation function calls *fe* as an iterative metric, we counted the call time share of different functions in MATLAB, as shown in Table 4, where Fitness is the fitness evaluation function, pdist2 is a function that calculates and ranks the distance between the IoT devices and the UAV. The result demonstrates the main function as well as the two functions with the highest run time share of DSLPSO for a particular run, which was a total of 119.035 seconds for this experiment.

**Table 4:** Statistics on the number of function calls and CPU time consumed.

Function	main	Fitness	pdist2
**Number of Calls**	1	1000416	1000416
**Running time (s)**	119.035	109.653	59.708
**Self-use time (s)**	7.688	49.945	46.316
**Percentage of time spent on own account**	6.46%	41.96%	38.91%
**Caller**	-	main	Fitness

The percentage of computation of the fitness evaluation function to the overall amount of computation is 92.12%, and the computation of the process of finding the correspondence between the IoT device and the stopping point accounts for about half of the computation in the fitness evaluation function. From this perspective, it is reasonable to use the number of calls to the fitness evaluation function as an iteration metric.

### 4.3 Comparison of DSLPSO with other algorithms on energy consumption

In this phase, we compare the DSLPSO algorithm with a wide range of other algorithms in this research area, which is used to verify the performance of the algorithm proposed in this paper. The comparison algorithms include five algorithms, DEEM [33], JADE [34], SSA [35], IDA [15], DEVIPS [17], and BSADP [18].

The running results of JADE, DEEM, and DEVIPS algorithms are obtained from reference [17], the running results of SSA are obtained from reference [35], and the running results of BSADP are obtained from reference [18], and the comparisons are summarized in Table 5, where the results of the DSLPSO algorithm proposed in this paper are the averages of 100 runs.

**Table 5:** DSLPSO algorithm and other algorithms of the same type comparison of energy consumption.

*m*	JADE	DEEM	SSA	DEVIPS	BSADP	IDA	DSLPSO
100	1.4837E+06	1.3507E+06	1.2508E+06	1.2525E+06	1.2492E+06	1.2695E+06	1.2420E+06
200	2.9912E+06	2.7035E+06	2.5101E+06	2.5045E+06	2.5003E+06	2.5350E+06	2.4764E+06
300	4.3166E+06	3.8755E+06	3.5982E+06	3.5809E+06	3.5788E+06	3.6150E+06	3.5321E+06
400	6.0787E+06	5.3782E+06	5.0008E+06	5.0016E+06	4.9937E+06	5.0422E+06	4.9149E+06
500	7.4686E+06	6.5991E+06	6.1136E+06	6.1248E+06	6.1143E+06	6.1802E+06	6.0218E+06
600	9.2240E+06	8.0769E+06	7.5436E+06	7.5628E+06	7.5408E+06	7.6325E+06	7.4234E+06
700	1.0434E+07	9.1732E+06	8.5631E+06	8.5702E+06	8.5525E+06	8.6610E+06	8.3973E+06

The experimental results show that: The average energy consumption obtained by the DSLPSO algorithm is better than the other five algorithms on each test case. The advantage of DSLPSO algorithm over the other algorithms increases as the size of the data collection task increases.

In our experiments, we further investigated the DEVIPS and IDA algorithms, in which the DEVIPS algorithm solves the results with a high probability of not being locally optimal (We will show in Section 4.4), and the IDA algorithm adopts the seeding radius strategy to limit the movement range of the individuals, but the updating mechanism which is superior to that of the seeding radius is exponential, and therefore the radius will decrease very fast during the optimization process, which results in the IDA algorithm not having a good ability to perform global searches.

In addition, we also recognize that the difference in the overall optimization results between DEVIPS, BSADP, SSA, IDA, and DSLPSO algorithms is not very large, and all of them can be regarded as effective algorithms in practical applications. Therefore, we are curious whether the optimization algorithms have reached a relatively optimal result for the current problem, and we have counted the results of the DSLPSO algorithm. The optimal result for 100 runs is shown in Table 6.

**Table 6:** Average and minimum values of the DSLPSO algorithm over 100 runs.

*m*	Mean	Min
100	1.2420E+6	1.2292E+6
200	2.4764E+6	2.4555E+6
300	3.5321E+6	3.5032E+6
400	4.9149E+6	4.8880E+6
500	6.0218E+6	5.9834E+6
600	7.4234E+6	7.3868E+6
700	8.3973E+6	8.3646E+6

It can be seen that the overall average is not much different from the optimal optimization gap, therefore, we believe that DEVIPS, BSADP, SSA, and the DSLPSO algorithm are all effective algorithms for the UAV mission planning, and the DSLPSO has the best performance.

**Fig 6:**
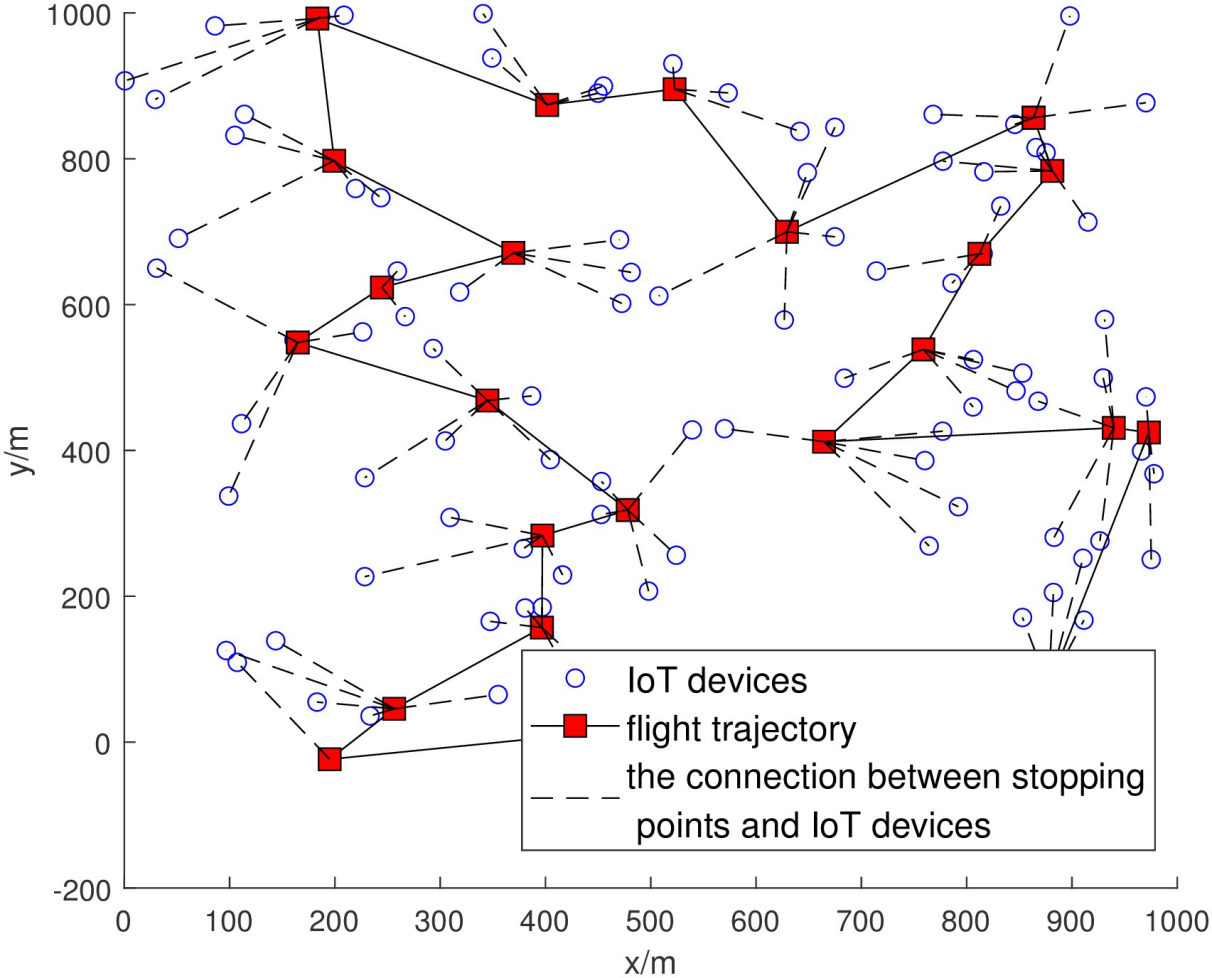
UAV trajectory example for *m* = 100.

Finally, we give the trajectory example of the UAV corresponding to one solution for *m* = 100 as shown in Fig 6 (We used LKH algorithm [36] to solved this trajectory).

### 4.4 Impact of region size changes and number of IoT devices changes

In this phase, we focus on the impact of the size of data collection task on results, using the DEVIPS algorithm as a comparison algorithm, we test the performance of the two algorithms in data collection tasks with different numbers of IoTs and different mission ranges, where *range* denotes the range size of the data collection task: *range* = *X*_*max*_ = *Y*_*max*_, the experimental results are shown in Table 7, in which each algorithm is executed independently 100 times for each test case.

**Table 7:** Optimization results of DEVIPS and DESLPSO in different experimental environments.

*m*	*range*	Algorithm	Mean	Std	Rate
100	1000	DSLPSO	1.2737E+6	5.4226E+3	0.000
100	1000	DEVIPS	1.2811E+6	5.6992E+3	0.955
100	2000	DSLPSO	1.2933E+6	8.2249E+3	0.000
100	2000	DEVIPS	1.3014E+6	8.2024E+3	1.174
100	3000	DSLPSO	1.2580E+6	6.3638E+3	0.000
100	3000	DEVIPS	1.2678E+6	5.9323E+3	1.783
200	1000	DSLPSO	2.3792E+6	1.0461E+4	0.000
200	1000	DEVIPS	2.4083E+6	9.4983E+3	0.681
200	2000	DSLPSO	2.4093E+6	1.0013E+4	0.024
200	2000	DEVIPS	2.4405E+6	1.0109E+4	1.122
200	3000	DSLPSO	2.2351E+6	9.7541E+3	0.044
200	3000	DEVIPS	2.2658E+6	1.0182E+4	1.432
300	1000	DSLPSO	3.7658E+6	1.0660E+4	0.015
300	1000	DEVIPS	3.8217E+6	1.4498E+4	1.029
300	2000	DSLPSO	3.3558E+6	1.0293E+4	0.092
300	2000	DEVIPS	3.4093E+6	1.5076E+4	1.406
300	3000	DSLPSO	3.5174E+6	1.2446E+4	0.106
300	3000	DEVIPS	3.5697E+6	1.4334E+4	1.235
400	1000	DSLPSO	4.8872E+6	1.2951E+4	0.023
400	1000	DEVIPS	4.9696E+6	1.7630E+4	0.800
400	2000	DSLPSO	4.8850E+6	1.1152E+4	0.080
400	2000	DEVIPS	4.9602E+6	1.4983E+4	1.272
400	3000	DSLPSO	4.9657E+6	1.2763E+4	0.239
400	3000	DEVIPS	5.0530E+6	1.7109E+4	1.418
500	1000	DSLPSO	6.3732E+6	1.4062E+4	0.081
500	1000	DEVIPS	6.4860E+6	2.0845E+4	0.949
500	2000	DSLPSO	6.1328E+6	1.4510E+4	0.202
500	2000	DEVIPS	6.2474E+6	1.7261E+4	1.385
500	3000	DSLPSO	6.0265E+6	1.3649E+4	0.436
500	3000	DEVIPS	6.1394E+6	2.2565E+4	1.542

The result shows that DSLPSO has lower average energy consumption in every test case of the experiment, with only a few cases performing slightly worse in terms of stability (i.e., standard deviation comparisons), so DSLPSO has greater generality and can be applied to tasks in different scenarios.

In order to verify the point that evolutionary algorithms such as DEVIPS, “the search ability is not strong in the local range” as we mentioned above (Section 3.2), we designed a “rate” indicator, which is a statistical value to measure whether the algorithms are optimized to a local optimum or not, and its implementation is as follows: traverse each UAV stopping point in the result of algorithm solving, move this point in four directions, up, down, left, right and 10 meters in each direction, and evaluate its adaptability, count the number of times that is generated due to these four kinds of movement, and “rate” is the average number of times, which can be seen that “rate” should be in the range of 0 to 4, and the smaller the value means that the algorithm is more effective in the local. From the results of “rate”, it can be seen that the DEVIPS algorithm is not as effective as the DSLPSO algorithm in local, and in many cases, its solution is not the local optimal solution, which is mainly due to the fact that the evolutionary algorithm is not very sensitive to the positional information when solving the current problem, but at the same time, its global search ability is still very good, and has better solution capability for problems of different sizes.

Looking at the relationship between energy consumption and range reveals that the variable range does not have as much influence on the experiment as we initially expected, and in the experiment, the variable range does not show a significant impact on the experimental results: e.g., for m = 100, the energy consumption of the DSLPSO algorithm for the solution at range = 1000, 2000 and 3000 are 1.2737E+6, 1.2933E+6 and 1.2580E+6 respectively, and the variation of the results of the DEVIPS is consistent with it, which implies that the distribution of IoT devices has a greater impact on the results under the current conditions. We believe that such results are reasonable due to the following two main reasons: The UAV flies at an altitude of 200, and the range of the task is not particularly large. In addition, we also counted the percentage of transmission energy consumption and hovering energy consumption, and we found that in most cases transmission energy consumption accounted for less than 5% of the overall energy consumption and that the total energy consumption in most cases consisted of the hovering energy consumption of the UAVs.

### 4.5 Impact of the number of MSA executions

The experiments in this section focus on the relationship between *qtime* and the results in our proposed MSA strategy.

In the process of studying the MSA strategy, we found that the number of IoT devices and the number of iterations (*feMax*) set for the algorithm have a certain effect on energy consumption, and the specific relationship between the effects is uncertain, to further study the relationship between the *qtime*, *feMax* and the size of the problem, we designed the present experiment.

We change the size of the problem by changing the number of IoTs, setting the number of IoTs as 100, 200, …, 600 for six sets of experiments, and the number of iterations for each set of experiments are 100k, 200k, …,600k, and *qtime* is 2,3,4,5 respectively, for a total of 6 × 6 × 4 = 144 experiments. The DSLPSO algorithm was run 10 times in each experiment. Some data of the experimental results are shown in Table 8.

**Table 8:** *qtime*, number of IoT devices, *feMax* effect on energy consumption (partial experimental results, the full results of the experiments can be found in the S1 Table).

*qtime*	Indicator	100,100k	100,200k	100,300k	200,100k	200,200k	200,300k
2	mean	1.2791E+6	1.2789E+6	1.2817E+6	2.3097E+6	2.2949E+6	2.2990E+6
	std	5.3809E+3	6.1552E+3	6.3736E+3	8.0107E+3	1.0445E+4	1.0518E+4
3	mean	1.2836E+6	1.2840E+6	1.2792E+6	2.3031E+6	2.2993E+6	2.2921E+6
	std	8.0886E+3	6.0824E+3	5.6995E+3	1.2384E+4	9.4275E+3	7.7480E+3
4	mean	1.2774E+6	1.2835E+6	1.2771E+6	2.3074E+6	2.2995E+6	2.3003E+6
	std	3.4114E+3	3.4915E+3	9.8202E+3	8.0799E+3	5.8099E+3	1.2511E+4
5	mean	1.2786E+6	1.2790E+6	1.2752E+6	2.3069E+6	2.3035E+6	2.2987E+6
	std	7.3549E+3	4.5728E+3	6.4399E+3	1.2876E+4	9.4745E+3	9.2798E+3

**Fig 7:**
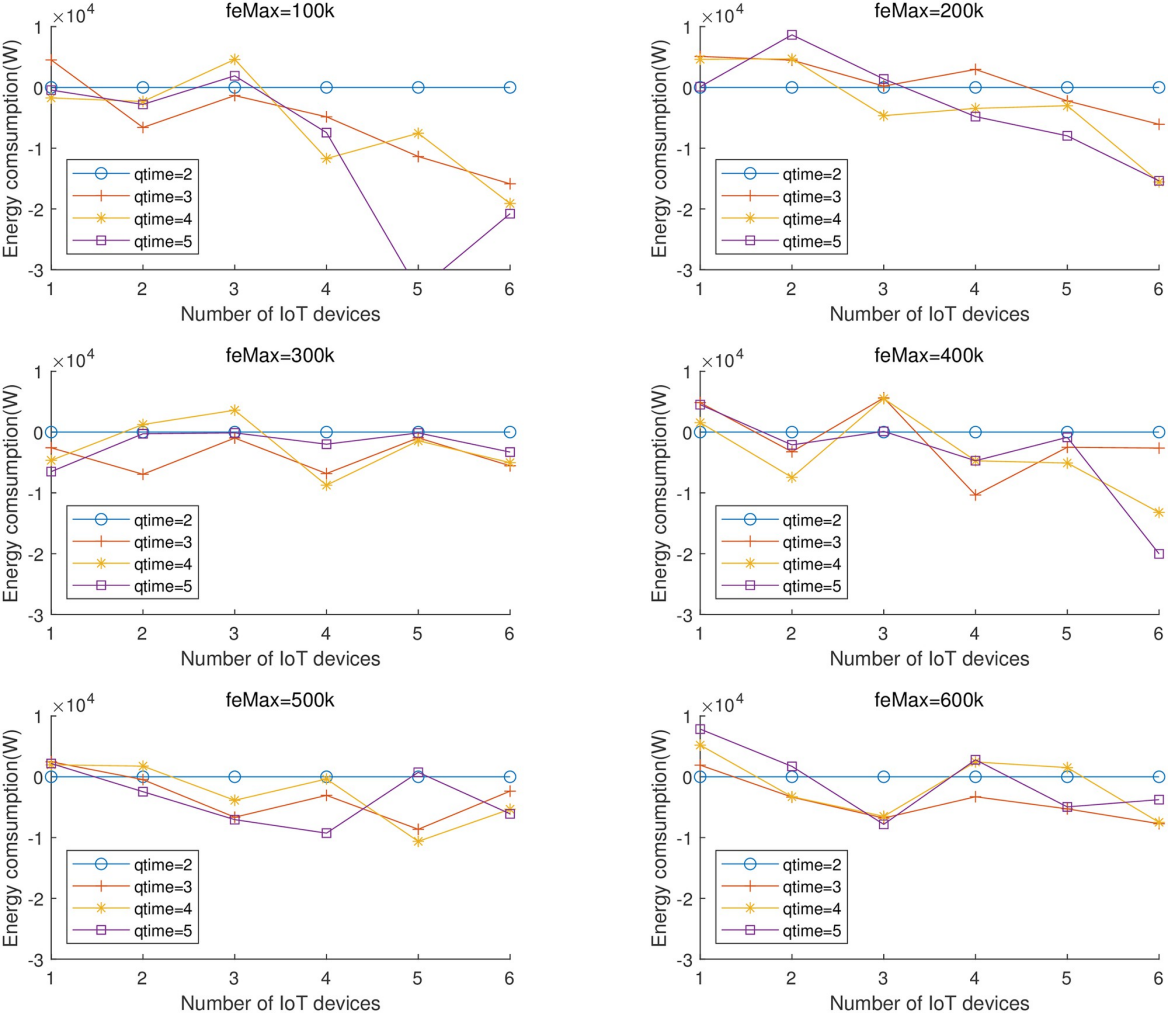
Relationship between energy consumption and number of IoT devices for different number of iterations with the benchmark of *qtime*=2.

Due to the relatively large amount of experimental data, the relationship between the number of annealing and the results cannot be clarified from the experiments in Table 8, for this reason we make the following considerations and plot the comparison figure: the most important purpose of this experiment is to study the relationship between the number of *qtime* and the problem complexity, but at the same time, we believe that the different number of *feMax* has a certain impact on the algorithms, so we consider the DSLPSO with different number of annealing as different algorithms, a total of 4 algorithms, fixing *feMax* and plot the effect of the performance of the 4 algorithms with the change of the number of IoTs, but this will face another problem: the difference in effect is not so significant (compared to the total energy consumption) when DSLPSO is chosen with different *qtime*, the order of magnitude of the energy consumption is large, and the plotted folds will be very close to each other, so we take DSLPSO algorithms with *qtime* = 2 as a baseline and the energy consumption results for the algorithms with *qtime* = 3, 4, 5 minus the energy consumption of the baseline plotted in Fig 7.

It can be seen from the figure: when the number of IoT devices is small (m = 100) using *qtime* = 2 is a better strategy, and as the number of IoTs rises, an increase in *qtime* helps to improve the effectiveness of the algorithm. We believe that: as the problem complexity and the number of iterations increase, an appropriate increase in *qtime* could help to improve the performance of the algorithm. When *feMax* > 300, increasing *qitme* does not change much since the optimization reaches its limit.

## 5 Conclusion

In the UAV-based data collection system, the optimization of the problem is highly challenging because the number and position of the stopping points of the UAV are unknown. In this study, we refer to the motion patterns of PSO and propose the SLR mechanism and MSA strategy to improve the algorithm’s ability to local and global search.

Genetic algorithms, such as DEVIPS algorithm [17] have a strong global search capability, but the local search capability is not strong due to the genetic inheritance among individuals, swarm algorithms, such as IDA algorithm [15] have a strong local search capability, but the updating strategy of the seeding radius is too fast, resulting in a lack of global search capability, whereas, our proposed SLR mechanism enhances the algorithm’s local search capability and ensures the algorithm’s local search capability by using the MSA strategy.

We found that there are some current studies on sensing networks focusing on data latency [30], sensor lifetime [31], and wireless transmission protocols [32]. In our next study, we will discuss these three issues based on the current research using environmental monitoring as an application environment to construct an automatic monitoring framework.

## Supporting information

S1 Table Result for impact of the number of MSA executions. Full results of the experiment.
(XLSX)

## Abbreviations

The abbreviations covered in this paper are shown in below.

PSO: Particle swarm optimization
DSLPSO: Double self-limiting PSO
IoT: Internet of things
SLR: Self-limiting radiu
MSA: Multiple simulated annealing
DEVIPS: Differential evolutionary algorithm for variable population size
BSADP: Backtracking search algorithm with dynamic population
SAPSO: Self-adaptive PSO
VPPSO: Variable population PSO
DEEM: Differential evolution with a new encoding mechanism
JADE: Adaptive differential evolution with a small population
SSA: Salp swarm algorithm
IDA: Improved dandelion algorithm
LKH: Lin-Kernighan traveling salesman heuristic algorithm
